# Understanding, being, and doing of bioethics; a state-level cross-sectional study of knowledge, attitude, and practice among healthcare professionals

**DOI:** 10.1186/s12910-024-01028-w

**Published:** 2024-03-18

**Authors:** Poovishnu Devi Thangavelu, Balamurugan Janakiraman, Renuka Pawar, Pravin H. Shingare, Suresh Bhosale, Russel D Souza, Ivone Duarte, Rui Nunes

**Affiliations:** 1 Center of Bioethics, Faculty of Medicine, University of Porto, Porto, Portugal; 2 Krishna College of Physiotherapy, Krishna Vishwa Vidyapeeth (Deemed to be University), Karad, Maharashtra 415539 India; 3 International Chair in Bioethics, Krishna Vishwa Vidyapeeth (Deemed to be University), Karad, Maharashtra 415539 India; 4 SRM College of Physiotherapy, Faculty of Medicine and Health Sciences, SRM Institute of Science and Technology (SRMIST), Kattankulathur, Tamil Nadu 603203 India; 5 School of Dental Sciences, Krishna Vishwa Vidyapeeth (Deemed to be University), Karad, Maharashtra 415539 India; 6 Pro Chancellor, Krishna Vishwa Vidyapeeth (Deemed to be University), Karad, Maharashtra 415539 India; 7 Krishna Vishwa Vidyapeeth (Deemed to be University), Karad, Maharashtra 415539 India; 8 International Institute of Organizational Psychological Medicine, Melbourne, Australia; 9 Center of Bioethics, Faculty of Medicine, and MEDCIDS - Department of Community Medicine, Information and Decision in Health; Faculty of Medicine, University of Porto, Porto, Portugal; 10 Center of Bioethics, Faculty of Medicine, University of Porto, Porto, Portugal

**Keywords:** Bioethics, Healthcare professionals, Knowledge, Attitude, Practice, Maharashtra, India

## Abstract

**Background:**

The field of bioethics examines the moral and ethical dilemmas that arise in the biological sciences, healthcare, and medical practices. There has been a rise in medical negligence cases, complaints against healthcare workers, and public dissatisfaction with healthcare professionals, according to reports from the Indian Medical Council and other healthcare associations. We intend to assess the level of knowledge, attitude, and practice of bioethics among the registered healthcare professionals (HCPs) of Maharashtra, India.

**Methods:**

A State-level online survey was conducted among the registered HCPs (*n* = 2143) casing all five regions of the Maharashtra state using a pre-tested self-administered questionnaire. The responses were expressed as mean, and proportions with their standard deviation and 95% CI respectively. Binary logistic regression and a multivariate logistic model were used to determine factors associated with knowledge, attitude, and practice of bioethics.

**Results:**

Of the 2143 registered HCPs in Maharashtra included in this study, most of them (65.2%) had adequate knowledge of bioethics. Adequate knowledge was associated with lower age, profession (nurses and dentists), employment in the private sector, HCPS at Marathwada and Pune, and higher educational attainment. About 3 in 5 HCPs (59.4%) had a favorable attitude towards the ethical practice of bioethics, and was associated with profession, place of work, region of practice, and work experience. The distribution of unethical bioethics practices among 10 items was proportionally high, and only 34.4% reported good/fair practice. The common unethical practices in the state were allowing patients to be examined by interns, and not informing them about professional misconduct to the regulatory bodies.

**Conclusion:**

Most HCPs had adequate knowledge of bioethics, which is encouraging and would favor the laying foundation for forming a good bioethics framework. Only 3/5 HCPs demonstrated a favorable attitude, and the observed unethical practice is alarmingly common. A serious consideration to evaluate the compliance level of bioethics practice periodically and measures to educate, sensitize, and train bioethics among HCPs in Maharashtra is warranted.

**Supplementary Information:**

The online version contains supplementary material available at 10.1186/s12910-024-01028-w.

## Background

Bioethics is a comprehensive term referring to the study of moral issues that transpire in the practice of medicine, health care, and biological sciences. It is inclusive of sub-domains like clinical ethics, professional ethics, and ethics of public policy [1, 2]. India is a country with a rich culture and diverse population and traditional ethics and cultural ethical values may interface with the principles of bioethics in such societies, where HCPs may not readily receive and practice existing principles of bioethics [3]. Middle and low-income countries cannot afford to oversee the discrimination, human rights violations, and injustice, particularly with the increase in medical negligence cases, deterioration of patient-doctor relationships, and loss of faith in the medical fraternity [4]. The advancement of the healthcare system, technological advancements, and inflation of cost have resulted in ethical dilemmas among health workers in most Asian countries. The education of Bioethics in India is mostly developed and conducted in the academic context of medical and nursing education, which is usually presented as reading and lecturing. A cognitive knowledge-centric skills approach among clinical students tends to result in neglecting attitude and character development, and beforehand decision-making without providing room for case-based moral questions, among future healthcare professionals [5, 6].

The National Consumer Disputes Redressal Commission (NCDRC), India reports that the increase in medical negligence is attributed to a lack of awareness of medical knowledge of ethics, rights of patients, poor moral values, and a rise in medical cost [7, 8]. India is a populous country with diverse ethno-cultural religious people across the regions and states, and the challenges of proper health care delivery are high. The Indian Medical Association (IMA) reported that about 75% of doctors faced violence at work and most of them (50%) from patients. Whilst, the recent rise in the reporting of medical negligence, public dissension against health care professionals, private, and public institutions, and registered complaints against health care workers in Maharashtra is an alarming sign of possible breaches of one or more of the Bioethics principles in medical practice [9, 10]. The Maharashtra state with 124 million people has the largest Health Delivery System (HDS) in the country and it is high time to explore the understanding and doing of Bioethics in clinical practice among HCPs of Maharashtra to set forth quality health care, and human values along with Sustainable Development Goals, India (SDG 3) motto of health for all [11, 12].

Previous studies conducted in India, Sri Lanka, Nepal, Bangladesh, Saudi Arabia, and Ethiopia with relatively smaller sample-based surveys reported that the proportion of poor knowledge of bioethics among physicians ranged from 39% to 81.2% [13–19]. And, concerning the proportion of physicians in South Asia (12% to 41%) reported occasional unethical practices related to drug prescriptions, accepting gifts from drug manufacturers, and obtaining leave [13–16]. Studies conducted in neighboring countries have observed the deteriorating standards of medical service and commercialization of healthcare services, which could easily tamper the ethics and human values. The most common unethical practice reported in South Asian countries is the recruitment of agents to refer patients, advertising, unnecessary surgeries, prescribing medication in the interest of manufacturers, and overcharging of fees [14, 16, 19, 20].

Although there are growing reports of biases that can distort the bioethics work, and the knowledge, attitude, and practice of bioethics by the researchers, and Health care professional (HCPs) play a vital role in achieving the vision of quality healthcare, it has surprisingly received fragmented attention compared to other fields in India. Despite the previous Indian studies prompting a substandard KAP of bioethics among a small proportion of HCPs, the evidence are based on less powered samples and majority of the studies reported [21–25] KAP-bioethics among medical students or residents. Further, there are hardly any studies describing the ethical practice of clinicians at the community level in India including Maharashtra. Research on the KAP of bioethics is crucial to strengthen the evidence and organize responsible interventions. In the background of reports from the IMA and NCDRC on rising rates of violence against medical professionals and medical malpractice, this study was designed to determine the level of knowledge, attitude, and practice of bioethics, and describe factors associated with KAP of bioethics. Specific objectives were to: Assess the knowledge, attitude, and practice of bioethics, and investigate the relationship between the socio-demographic, and work-related characteristics of participants with KAP of bioethics.

## Methods

### Study design, period, setting, and participants

A state-wide online survey study was conducted using the cross-sectional analytical design from January 2023 to April 2023. The study protocol (no: 395/2020-2021) was approved by the University of Porto, presented to and approved by the Ethical board Krishna Viswa Vidyapeeth (KVV), Maharashtra, India (ref no KIMSDU/IEC/06/2021). This study included Healthcare professionals (HCPs) involved in direct patient care. The occupational categories that fit our definition of direct patient care included; physicians, dentists, nurses, physiotherapists, and occupational therapists.

Maharashtra is the largest state economy and 2^nd^ most populous in India. Maharashtra has a total population of 112.4 million with a projection to increase to approximately 124 million by 2020 and formed 9.28 percent of India in the 2011 Census. The literacy rate of the state is 82.34%, which is greater than the literacy rate (72.98%) of India and the sex ratio of the state is 929 per 1000 men [11, 12]. According to the Asian Development Bank (ADB), the Maharashtra government facilities suffer from a lag of healthcare providers (148 doctors and nurses in public hospitals for every 100,000 population), limited specialists, overcrowding, and poor quality of service [26]. According to the National Health Systems Resource Centre (NHSRC) reports (2021) the per capita Government Health Expenditure in the state was 1,356 Indian rupees, which was 33% less than the national average. In public health facilities, the percentage of money spent on medications for inpatient care is estimated to be 41% in rural areas and 36% in urban areas, respectively; in contrast, the percentage for diagnostics is approximately 22% and 11% in both areas, respectively [27]. The health infrastructure report indicated that the state has 49 district hospitals, 100 sub-district hospitals, 26 government teaching hospitals, and 31 private teaching hospitals. The Sustainable Development Goals 2030 (SDGs India Index, NITI Aayog) tracks state-wise progress which reported that the SDG-3 (good health and well-being) index score of Maharashtra is 60 out 100. The SDG Index Score for the Goal of Good Health and Well-being ranges between 25 and 92 for 7 larger states of India. Maharashtra is categorized as a “performer” by the NITI Aayog which is “average” in terms of national performance [28]. Several principles of bioethics apply to moral issues and actions related to SDGs bringing conscience to society. Ethics and SDGs are interconnected, there is a correlation between the main characteristics of ethics with the objectives of SDGs. For instance; justice (SDG 16), equity in health and non-discrimination (SDG 3.8, SDG 5, and SDG 8), and non-maleficence (SDG 3) [29].

All the HCPs registered in the Maharashtra Medical Council, Maharashtra Nursing Council, Maharashtra Dental Council, Maharashtra Physiotherapy Council, and Maharashtra Occupational Therapist Council with a minimum of 1-year service in patient care in the Maharashtra state, and currently practicing in the state were the source population. Participants who had registered with the state council but not in patient care practice currently, not residing or practicing in Maharashtra, overall service year of less than one year were instructed not to respond to or submit the online bioethics KAP survey form. This study was reported according to the STROBE (Strengthening the Reporting of Observational Studies in Epidemiology) guidelines [30]. Health care professionals [31] are defined as those who are qualified clinicians (physicians, dentists, physiotherapists, occupational therapists) and nurses registered with the Maharashtra state government, and involved in patient care for at least 30 hours a week for a minimum of one year.

### Sample size determination and sampling

A multi-centered State-wise (Maharashtra, India) online survey was conducted and the power calculated sample was determined based on the following assumptions [32] using the formula for single population proportion; for infinite population (Registered HCPs practicing in the state), with no valid regional studies and based on the recommendation for sample size assumptions [33], we assumed that 50% prevalence (p) of the population to have adequate knowledge, favorable attitude, and good practice of bioethics, considering a 95% confidence interval, and 5% precision. Further, the variance of the binomial distribution is n*p*(1-p) is maximum at 50% value of ‘p’ for any given ‘n’. The derived sample size was 384. To allow a power representation from all 5 regions of the state, 384 samples from each region were proposed and the total sample required for all 5 regions was 1920. Presuming the higher non-response rate due to the online survey data collection methods, a required sample size was inflated by 50 % to cover the non-response rate, attrition, and contingency. The final derived sample was *n* = 2880. The authors managed to collect 3247 email IDs of health professionals from the Maharashtra State. The English version of the fillable online survey Google form (Google LLC, CA, USA) was sent to a total of 3000 email addresses which were collected from the registered bodies of respective professions, google from blog sites, and NGO (Healthcare) websites operating in Maharashtra, India after removing duplicate addresses. The authors decided to wait for 45 days to compile the responses, at this point, we received 1866 responses and to improve the response rate a reminder mail was sent on the 46^th^ day to those who did not reply, after which we received an additional 379 responses were received. So, in total we received 2245 filled-in survey Google forms. Responses received after 15 days of the reminder mail were not considered for data analysis.

### Outcome tool and variable definitions

A literature and guidelines-based structured questionnaire [34–36] was developed using a conceptual model and then items were generated. A focus group of experts including all the trained healthcare professionals in the field of bioethics were involved in the rating, modifying, and selecting items for assessing knowledge, attitude, and practice (KAP) in bioethics. The items in the questionnaire represented the following domains; autonomy, non-maleficence, dignity, beneficence, justice, confidentiality, benefits, non-discrimination, and vulnerability. The English version of KAP-Bioethics requested responses on socio-demographic, educational, and work characteristics, their knowledge, attitude, and practice concerning bioethics (S1 file). The KAP-Bioethics questionnaire was pretested by one one-week interval period test-retest method among 25 HCPs at KVV. The knowledge domain contained 10 questions with 4 choices including the correct answer, and the correct answer was scored ‘1’ and the wrong choices were scored ‘0’. The attitude and practice domains had 11 items each, 10 items each regarding attitude and practice towards bioethics, and 1 item each in attitude and practice were included to verify the attention of the respondent.

The questions in attitude and practice domains contained 5 Likert scale responses (strongly agree, agree, neutral, disagree, and strongly disagree). The strongly agree and agree responses were scored ‘1’, and neutral, disagree, and strongly disagree were scored ‘0’. Participants who answered correctly to all 10 knowledge questions, or scored more than, and equal to the mean value (6.96) were defined to have adequate knowledge about bioethics. Participants who responded positively (strongly agree or agree) to all 10 attitude-related questions and practice questions, or scored more than, and equal to the mean value (attitude 8.3 and practice 7.44) were defined to have a positive attitude and good practice towards bioethics.

### Development, validity, and reliability of the survey instrument

A conceptual framework was developed using an extensive literature review, norms referring to bioethics in India, and the focus group (16 expert members; 3 medical officers, 2 (bioethics chair) academic professors, 3 dentistry professors, 4 physiotherapists, and 4 nursing professors) evaluated the face and content validity of the English version of KAP-bioethics tool. A pre-test was conducted among 25 randomly selected HCPs from KVV from 5 (medicine, dentistry, PT, OT, and nursing) departments. The intra-class coefficient of the overall tool was 0.892 (95% CI 0.81, 0.92), the data for test-retest reliability was recorded with a 48 hours interval time, and the internal consistency (Cronbach’s alpha α) coefficient for knowledge, attitude, and practice were 0.73 (satisfactory), 0.80 (good), and 0.81 (good) respectively (S2 file). The Cronbach’s alpha (α) for deleting any item in the domain did not alter the score [37].

### Data management and analysis

The data were entered, cleaned, coded, and analyzed using Epi-data version 3.5 for Windows and the IBM Statistical Package for Social Sciences (SPSS, version 25.0 for Windows), IBM Corp, Armonk, NY, USA. The independent (KAP scores) and predictor variables were categorical (either by transforming or originally) and were described by mean, standard deviation, frequency distribution, and proportions with their 95% confidence interval. The knowledge (10 items) questions were scored (correct answers ‘1’ and incorrect answers ‘0’). The 10 items on the bioethics attitude were assessed using a 5-point Likert scale (coded as; strongly agree/agree ‘1’ and neutral/disagree/strongly disagree ‘0’). Bioethics variables related to knowledge, attitude, and practice were treated in two modalities. The pretest data of the survey tool was analyzed using the Intraclass correlation coefficient, the ICC value of ≥ 0.50, and Cronbach’s alpha (α) coefficient ≥ 0.70 were considered satisfactory for the test-retest reliability and internal consistency respectively. Binary logistic regression model with a cut-off *p*-value of 0.2 was used to identify the association between the outcome variable (KAP-Bioethics), and predictor variables. A stepwise approach was used to examine the association of KAP with independent variables that were significant in the univariate test in the multivariate model. The level of significance was set as 0.05, and the adjusted odds ratio with a 95% confidence interval was presented. The model fit for regression entry was assessed by the Hosmer and Lemeshow’s goodness of fit test [38], the results were considered significant if the 95% CI not containing unity (equal to *p*-value <0.05).

## Results

A total of 2245 responses were received with a response rate of 74.83%. The filled-in survey questionnaire containing inappropriate responses for one or both attention questions and incomplete responses were excluded (*n* = 102) and finally, 2143 responses were taken for further analysis. This (*n* = 2143) is more than the overall power calculated sample size for this study. The results are presented as follows.

### Socio-demographic and educational-work profile of the participants

Among the 2143 respondents, 940 (43.9%) were men and 1203 (56.1%) were women. The mean age of the respondents was 41.76 ± 8.9 years. Medical doctors and nurses registered with the state council predominated, representing respectively 40.4% (866/2143), and 41.5% (889/2143), followed by dentists 11.75 (250/2143). Physiotherapists 5.5% (117/2143), and occupational therapists 1% (21/2143). Most of the respondents in this study were from private 56.1% (1203/2143) and government institutions 42.6% (912/2143). Near about 2/3^rd^ (66.1%) of the respondents had post-graduation degrees, and only 0.2% (05/2143) super specialists participated in this study. Almost regional state-wise proportional respondents from Konkan (16.4%), Marathwada (19.2%), Nashik (19.4%), Pune (25.3%), and Vidarbha (19.7%) participated. The participants had a mean work experience of 15.67±7.26 years. The majority of the HCPs (58.9%, 1261/2143) reported having more than 15 years of work experience (Table 1).

**Table 1:** Socio-demographic characteristics of the study participants (*n* = 2143)

**Variables**		**N**	**%**
Age (years)	< 35	546	25.5
mean 41.76±8.9	35 – 50	762	35.6
median 41.0, IQR 34,49	>50	835	39
Sex	Male	940	43.9
	Female	1203	56.1
Health profession	Physician	866	40.4
	Nurse	889	41.5
	Dentist	250	11.7
	Physiotherapist	117	5.5
	Occupational therapist	21	1.0
Work place	Governmental	912	42.6
	Private	1203	56.1
	Others (NGO)	28	1.3
Qualification	Under graduate	721	33.6
	Post-graduate	1417	66.1
	Super-specialty	05	0.2
Region of practice	Konkan	351	16.4
	Marathwada	412	19.2
	Nashik	415	19.4
	Pune	543	25.3
	Vidarbha	422	19.7
Work experience (years)	< 5	279	13
mean 15.67±7.26	5 –10	209	9.8
	11 – 15	394	18.4
	16 – 20	629	29.4
	>20	632	29.5

± Standard deviation, *IQR* Inter-Quartile Range, *NGO* Non-governmental Organization

### Knowledge, attitude, and practice of bioethics among health care professionals

Based on the 10-item knowledge domain questions, 65.2%, (95% CI 63.2, 67.1, *n* = 1398/2143) of the participants were observed to have adequate knowledge of bioethics. The mean knowledge score of bioethics among the HCPs of Maharashtra state was 6.96 (± 1.2) and ranged from 2 to 9. About 91.5%, 86%, and 81.7% of HCPs correctly identified the items K8, K4, and K1 respectively. Similarly, 65.5%, 46.2%, and 47.3% of respondents were found to have inadequate knowledge regarding the items K9, K6, and K7 respectively (Table 2). The frequency distribution of adequate knowledge among different HCPs observed higher bioethics knowledge among nurses (80.8%, 718/889), physicians (76.3%, 661/866), and dentists (5.2%, 188/250) with a near similar mean score of 7.3, 7.1, and 6.7 respectively (Table 3).

**Table 2:** Knowledge of bioethics among healthcare professionals based on choice questions with one possible answer, representing the core principles of bioethics (*n* = 2143)

**Item Code**	**Knowledge questions**	**Response**	
		**Appropriate n (%)**	**Inappropriate n (%)**
K1	Paternalism is not an ethical attitude because it conflicts with	1751 (81.7)	392 (18.3)
K2	Which of the following is not true about non-maleficence	1689 (78.8)	454 (21.2)
K3	Double effect in bioethics is regarded as the combined effect of	1495 (69.5)	648 (30.5)
K4	The concept of justice in Bioethics is	1842 (86)	301 (14)
K5	Confidentiality can be breached	1666 (77.7)	477 (22.3)
K6	Right versus wrong may be a moral temptation, but right versus right is	1152 (53.8)	991 (46.2)
K7	Specific code or metaprogram hard-wired in the human mind that can be exploited to cause harm is	1130 (52.7)	1013 (47.3)
K8	The most important advantage of an advance directive is to	1961 (91.5)	182 (8.5)
K9	Informed consent is a	739 (34.5)	1404 (65.5)
K10	The Institutional review board (IRB) is charged with?	1492 (69.6)	651 (30.4)
Total K score, mean ±SD		6.96±.1.2	
**Summary index of Bioethics Knowledge**			
Adequate knowledge, n (%), 95 CI		1398 (65.2%), 95% CI 63.2,67.1	
Inadequate knowledge, n (%), 95 CI		745 (34.8%), 95% CI 32.9, 36.8	

(95% CI) Confidence Interval

**Table 3:** Distribution of knowledge score across the healthcare professionals (*n* = 2143)

**Healthcare profession**	**Total,** **n (%)**	**Response to knowledge**		
		Mean ±SD	Adequate n (%)	Inadequate n (%)
Physician	866 (40.4)	7.1 ± 0.99	661 (76.3)	205 (23.7)
Nurse	889 (41.5)	7.3 ± 1.12	718 (80.8)	171 (19.2)
Dentist	250 (11.7)	6.71 ± 1.15	188 (75.2)	62 (24.8)
Physiotherapist	117 (5.5)	6.24 ±1.12	46 (39.3)	71 (60.7)
Occupational therapist	21 (1.0)	6.75 ± 1.7	15 (71.4)	06 (28.6)

#### SD – Standard deviation

The proportion of HCPs who had a favorable attitude towards bioethics was 59.4% (95% CI 57.4, 61.5). A higher proportion of the HCPs reported having a favorable judgment towards the attitude item; the importance of ethical conduct to avoid legal actions (item A11, 79.2%), and refusal of treatment by patients due to belief (item A2, 78.5%) (Table 4). Most HCPs (80.4%) expressed neutral stands toward the need for performing multiple tests to avoid medical errors. About one-third of HCPs disagreed with the referral of patients during unsure diagnosis.

**Table 4:** Attitude towards bioethics among participants (*n* = 2143)

**Item**	**Attitude questions**	**SA** **n (%)**	**A** **n (%)**	**N** **n (%)**	**D** **n (%)**	**SD** **n (%)**
A1	If the diagnosis of the referred patient is unsure better refer to the expert physician	470 (21.9)	899 (42)	7 (0.3)	706 (32.9)	61 (2.8)
A2	If patients refuse treatment due to beliefs, they should be directed to find another healthcare professional	1682 (78.5)	375 (17.5)	2 (0.1)	2 (0.1)	82 (3.8)
A3	All official documents relating to patient care need authenticity with the health professional identity particulars duly signed.	1266 (59.1)	643 (30)	174 (8.1)	7 (0.3)	53 (2.5)
A4	A healthcare professional can legally disclose information to protect the patient from danger	1189 (55.5 )	556 (25.9)	342 (16)	3 (0.1)	53 (2.5)
A6	An emotional or sexual relationship with a patient (or with a member of the patient’s family), even with consent, is unethical	1290( 56.4)	879 (41)	9 (0.4)	5 (0.2)	41 (1.9)
A7	After the successful completion of research, its benefits should be shared to society globally.	997( 46.5)	930 (43.4 )	11 (0.5)	171 (8)	34 (1.6)
A8	A male healthcare professional should refuse to examine an uncomfortable female patient without a female attendant.	709 (33.1)	1105 (51.6)	194 (9.1)	106 (4.9)	29 (1.4)
A9	Multiple tests can be performed to avoid a medical error	10 (0.5)	203 (9.5)	1722 (80.4)	180 (8.4)	28 (1.3)
A10	The patient needs to be informed about their diagnosis even if they don’t have the requisite knowledge	659 (30.8)	1255 (58.6)	199 (9.3)	7 (0.3)	23 (1.1)
A11	Ethical conduct is important for avoiding legal action	1698 (79.2)	395 (18.4)	12 (0.6)	6 (0.3)	32 (1.5)
**Summary Index of Attitude towards Bioethics**						
Attitude, positive n (%), 95% CI		1274 (59.4), 95% CI 57.4, 61.5				
Attitude, negative n (%), 95% CI		869 (40.6), 95% CI 38.3, 42.5				

Question A5 is an attention check item, hence not included in the analysis, *SA* Strongly agree, *A* Agree, *N* No opinion, *D* Disagree, *SD* Strongly disagree, *CI* Confidence Interval

The responses to the 10 statements regarding bioethics practice among HCPs are shown in (Table 5). Overall, only 34.4% (95% CI; 32.3, 36.4) expressed good or fair practice of bioethics. Accepting gifts or taking benefits from the patients was recognized as an unethical practice by 68.2% of HCPs. The proportion of HCPs reported to follow good bioethics practice was only 11.5%.

**Table 5:** Frequency distribution of responses for the practice of bioethics among the participants (*n* = 2143)

**Item**	**Practice questions**	**Always** **n (%)**	**Most often** **n (%)**	**Sometimes n (%)**	**Rarely** **n (%)**	**Never** **n (%)**
P1	I explain the nature, purpose, and possible consequences of treatment or procedure while obtaining informed consent from patients	831 (38.8)	1192 (55.6)	79 (3.7)	04 (0.2)	37 (1.7)
P2	During clinical rounds along with clinical aspects of patients care, I discuss ethical issues also	235 (11)	1139 (53.1)	733 (34.2)	10 (0.5)	26 (1.2)
P3	I practice equity especially when it is applied to resource management	250 (11.7)	1164 (54.3)	704 (32.9)	12 (0.6)	13 (0.6)
P4	I render the same level of care to my clients in regular practice and over-time	307 (14.3)	1543 (72)	265 (12.4)	10 (0.5)	18 (0.8)
P6	I regularly re-examine the patients to find the effectiveness of the ongoing treatment	1028 (48)	770 (35.9)	10 (0.5)	301 (14)	34 (1.6)
P7	I accept the patient’s request not to be examined by interns/trainees	188 (8.8)	1115 (52)	46 (2.1)	641 (29.9)	153 (7.1)
P8	I patiently listen to all the past history of the patient even if that doesn’t help the present treatment	589 (27.5)	1003 (46.8)	422 (19.7)	107 (5.0)	22 (1.0)
P9	Getting any form of benefits from patients treated by me is unethical	1462 (68.2)	505 (23.6)	142 (6.6)	02 (0.1)	32 (1.5)
P10	When Health records are shared with other doctors for opinion I secure it with suitable software security.	427 (19.9)	1292 (60.3)	292 (13.6)	108 (5.0)	24 (1.1)
P11	When I come across an instance of professional misconduct, I bring it to the notice of the regulatory authority	255 (11.9)	645 (30.1)	1199 (55.9)	12 (0.6)	32 (1.5)
**Summary Index of Bioethics Practice**						
Good/fair practice, n (%), 95% CI		737 (34.4), 95% CI 32.3, 36.4				
Bad Practice, n (%), 95% CI		1406 (65.6), 95% CI 63.6, 67.7				

Question P5 is an attention check item hence not included in the analysis

#### Determinants of KAP of bioethics

In the multivariate model, when adjusted for the other predictor variables, young age, health profession category, place of work, level of qualification, region of practice in Maharashtra, and lesser work experience were significant (*p* < 0.05) predictors of the knowledge about bioethics Table 6. The participants aged less than 35 years were found to have 1.5 folds (95% CI 1.13, 3.5) more knowledgeable than their counterparts. Among the HCPs nurses were observed to have better knowledge (AOR 1.36, 95% CI 1.17, 2.59) in bioethics, and on the other hand, the dentists and physiotherapists were found to be less knowledgeable (AOR 0.71, 95% CI 0.50, 0.94 and AOR 0.47, 95% CI 0.16, 0.72 respectively). Professionals with higher educational attainment were observed to have better knowledge in bioethics (postgraduate; AOR 2.6 (95% CI, 1.9, 5.6) and super-specialty: AOR 1.5 (95% CI, 1.19, 3.21). Those who practiced in the Marathwada region (AOR 2.78, 95% CI 1.34, 6.3) and at Pune (AOR 1.30, 95% CI 1.09, 2.13) were found to have better knowledge of bioethics than their counterpart.

**Table 6:** Bi-variable and multivariable logistic regression analysis result of KAP towards bioethics and factors associated among healthcare professionals, Maharashtra, India (*n* = 2143)

**Variables**	**Knowledge**		**Attitude**		**Practice**	
Age (years)	COR(95%CI)	AOR(95%CI)	COR(95%CI)	AOR(95%CI)	COR(95%CI)	AOR(95%CI)
< 35	2.25 (1.77, 2.86)	1.5(1.13, 3.5)*	1.95 (1.17, 3.62)	1.44 (1.10, 3.02)	1.89 (1.53, 2.32)	1.54 (1.17. 2.03)*
35 – 50	1.318(1.01, 1.62)	1.01(0.61, 1.24)	1.20 (0.86, 2.31)	0.99 (0.56, 1.77)	0.96 (0.78, 1.26)	0.86 (0.62, 0.57)
>50	1 ref	1 ref	1 ref	1 ref	1 ref	1 ref
Sex						
Female	1.33 (1.11, 1.6)	1.20(0.68, 2.34)	1.61 (1.25, 4.02)	1.13 (0.87, 4.13)	1.21 (1.01, 1.46)	1.34 (1.09, 2.55)
Male	1 ref	1 ref	1 ref	1 ref	1 ref	1 ref
Health profession						
Physician	1 ref	1 ref	1 ref	1 ref	1 ref	**1 ref**
Nurse	1.75 (1.41, 2.37)	1.36(1.17, 2.59)*	1.87 (1.29, 3.26)	1.51 (1.09, 2.88)*	9.34 (2.39, 25.9)	3.61 (2.67, 4.71)*
Dentist	0.29 (0.19, 0.38	0.71(0.50, 0.94)*	2.81 (1.53, 6.41)	1.99 (1.27, 4.05)*	3.38 (1.34, 7.20)	1.49 (1.08, 2.21)*
PT	0.21 (0.11, 0.71)	0.47 (0.16, 0.72)*	0.32 (0.18, 0.76)	0.54 (0.21, 0.79)	0.16 (0.10, 0.49)	0.51 (0.27, 0.97)*
OT	0.15 (0.1, 0.54)	0.26 (0.12, 0.63)	0.40 (0.31, 0.87)	1.21 (0.55, 3.02)	0.23 (0.19, 0.66)	0.60 (0.13, 0.91)*
Work place						
Governmental	1 ref		1 ref	**1ref**	1 ref	**1 ref**
Private	0.59 (0.23, 0.89)	**0.33 (0.15, 0.69)**	4.12 (3.4, 4.94)	2.75 (1.19, 5.72)*	7.54 (2.41, 16.4)	5.1 (1.49, 27.6)**
Others (NGO)	0.90 (0.43, 1.20)	0.97 (0.81, 1.23)	4.43 (1.86, 10.53)	1.97 (1.69, 3.02)*	10.2 (2.4, 13.4)	3.9 (1.07, 22.4)**
Qualification						
Under graduate	1 ref	1 ref	1 ref	1 ref	1 ref	1 ref
Post-graduate	5.51(3.5, 13.8)	2.6 (1.91, 5.63)**	1.52 (1.08, 6.33)	1.69 (1.48, 4.57)*	0.40 (0.34, 0.49)	0.49 (0.12, 0.73)
Super-specialty	1.85(1.21, 2.71)	1.5 (1.19, 3.21)**	4.5 (2.64, 8.15)	3.20 (1.25, 7.81)*	0.92 (0.87, 1.99)	0.33 (0.08, 0.94)
Region of practice				*		
Konkan	0.39(0.28, 0.56)	0.76 (0.59, 2.01)	1.68 (1.34, 9.26)	1.05 (0.78, 1.66)	2.72 (1.07, 2.03)	1.64 (1.13, 2.95)
Marathwada	4.93(3.25, 7.57)	**2.78 (1.34, 6.3)**	1.37 (0.95, 2.33)	1.24 (1.04, 3.11)	1.94 (1.64, 3.71)	1.89 (0.98, 5.23)
Nashik	0.29(0.21, 0.39)	0.22 (0.10, 0.74)	6.94 (2.40, 11.21)	**4.01 (1.98, 12.37)**	0.31 (0.16, 0.69)	0.56 (0.13, 0.87)*
Pune	1.69(1.21, 0.39)	**1.30 (1.09. 2.13)**	2.73 (1.68, 9.13)	**1.84 (1.16, 3.96)**	0.45 (0.34, 0.70)	0.73 (0.20, 0.91)*
Vidarbha	1 ref	1 ref	1 ref	1 ref	1 ref	1 ref
Work experience						
< 5	3.67(2.5, 5.3)	**1.75 (1.41, 3.01)**	3.98 (1.29, 5.32)	**2.69(1.59, 2.95**	2.29 (1.69, 3.10)	1.49 (1.31, 2.99)*
5 –10	1.05 (0.87, 1.17)	0.56 (0.12, 0.97)	4.15 (3.28, 5.18)	**3.01 (1.17, 7.51)**	1.50 (1.06, 2.38)	1.48 (1.07, .09)*
11 – 15	1.25 (1.01, 1.36)	1.01 (0.45, 2.13)	1.74 (0.98, 1.54)	1.08 (0.58, 1.84)	4.04 (3.17, 5.66)	2.87 (1.96, 4.21)*
16 – 20	1.61 (1.10, 1.96)	1.19 (0.85, 1.69)	0.41 (0.20, 0.81)	0.33 (0.15, 0.79)	0.83 (0.60, 1.13)	0.97 (0.59, 1.62)
>20	1 ref	1 ref	1 ref	1 ref	1 ref	
Knowledge						
Adequate					2.78 (1.67, 3.51)	2.16 (1.45, 3.21)*
Inadequate					1 ref	1 ref
Attitude						
Positive					0.92 (0.18, 1.67)	0.97 (0.59, 1.62)
Negative					1 ref	1 ref

*AOR* Adjusted Odds Ration, *COR* Crude Odds Ratio, *PT* Physiotherapists, *OT* Occupational therapist, *NGO* Non-Governmental Organizations

^*^Significant at <0.05

^**^Significant at <0.001

The dentists and nurses of the Maharashtra state had a favorable attitude (AOR 1.99; 95% CI 1.27, 4.05 and AOR 1.51; 95% 1.09, 2.88) towards bioethics in comparison to the other HCPs. The participants who worked at private (AOR 2.75; 95% CI 1.19, 5.72) and NGO (AOR 1.97; 95% CI 1.69, 3.02) were about twice more likely to have a positive attitude towards bioethics than the governmental employees. HCPs (mostly physicians) with super-specialty qualifications reported to have three folds favorable attitude (AOR 3.2, 95% CI 1.25, 7.81), post-graduate participants were nearly about 1.5 times more likely (AOR 1.69, 95% CI 1.48, 4.57) to have a favorable attitude towards bioethics compared to those with under-graduate degree. HCPs at Nashik had the most favorable attitude (AOR 4.01, 95% CI 1.98, 12.37), followed by those at Pune who were near about twice as likely (AOR 1.84, 95% CI 1.16, 3.96). The participants with lesser experience were observed to have favorable attitudes (<5 years, AOR 2.69; 95% CI 1.59, 2.95 and 5-10 years, AOR 3.01, 95% CI 1.17, 7.51) in comparison with those with more than 10 years of experience.

The HCPs who were found to have adequate knowledge were twice more likely to be engaged in good bioethics practice. And, attitude categories demonstrated no association with the bioethics practice. Among the professionals the nurses were 3 times more likely to engage in good bioethics practice (AOR 3.61, 95% CI 2.67, 4.71), followed by the dentists who were near about 1.5 times (AOR 1.49, 95% CI 1.08. 2.21) more likely. The OT and PT professionals reported bad practices of bioethics (AOR 0.51, 95% CI 0.2, 0.97 and AOR 0.60 95% CI 0.13, 0.91 respectively) in comparison to the other professionals. The younger participants aged less than 35 years are 1.5 times more likely to be engaged in good practice. The professionals working in private institutions (AOR 5.1, 95% CI 1.49, 27.6) and NGOs (AOR 3.9, 95% CI 1.07, 22.4) reported to be engaged in good practice compared to the governmental workers. The HCPs practicing at Nashik (AOR 0.56, 95% CI 0.13, 0.87) and Pune (AOR 0.73, 95% CI 0.20, 0.91) had poorer bioethics practice than the other regions in the State (Table 6).

## Discussion

The health care delivery system (HCDS) has been increasing in India, both in significance and magnitude. Yet, according to the WHO 2021 report, India was way behind the recommended and desirable healthcare workers to population ratio [39]. Further, the major concerns are a substantial proportion of health workers are not adequately qualified allopathic physicians, lack of quality of delivery, standard care, unethical revenue targets of private hospitals, and skewed proportion of health care forces across states, public-private, and rural-urban sectors [40, 41]. The fast-expanding Indian HCDS requires appropriate ethical oversight, a requirement that the Government of India has duly acknowledged in its national laws and regulations for health [42]. However, to the best of our knowledge based on literature search, this is the first study in India to report knowledge, attitude, and practice of bioethics among different HCPs across all the five regions of the state with power-calculated sample for each region in Maharashtra, India.

The findings of this study revealed that a moderate proportion of respondents showed adequate level of knowledge (65.2%) and positive attitude (59.4%) towards bioethics in general, yet only a small proportion of HCPs reported good practice (34.4%). The response rate (77.9%) of the present study was comparable to relatively similar studies ranging from 62% to 82% [13–16, 19, 43]. Despite the large contingency plan (50%) accommodated for this study, this non-response rate may be due to lack of time (average questionnaire time 25 min), sensitive questions regarding medical practice, and non-active email ID [44]. In any case, among the registered HCPs in the state physiotherapist and occupational therapists were observed with a low response rate.

### The level of knowledge and factors associated with bioethics

This study found that 34.8% of the HCPs in Maharashtra had poor knowledge of bioethics. Studies conducted to determine the proportion of Indian HCP's knowledge about ethics ranged from 41% to 81%. Most of the studies in India reported poor levels of knowledge, indicating that there is a need for education and implementation of guidelines to improve the understanding of contents [13, 14, 25, 43, 45]. The wide variance in the level of knowledge among the HCPs in India could be due to the difference in the study population, strata of a particular profession, rural-urban practice area, and working sectors (teaching hospitals, private and public hospitals) for example dentist practitioners, dental faculties, budding family physicians, doctors, and nurses. Most studies reporting knowledge, attitude, and practice of ethics in India included healthcare students and residents[13, 25]. However, the findings of this study are subjected to over or underestimation of responses due to the design of the study, and at the same time, the adequate level of knowledge does not guarantee good ethical conduct due to the self-reported nature of this study.

The studies conducted in South Asian countries, Sri Lanka, Pakistan, Nepal, and Manipur (India) found that 81.2%, 66%, and 70% had poor levels of knowledge on ethics [13, 15, 16, 19]. The authors of these studies used code of ethics-related questions from the medical council of their respective nations and the method of survey was a self-administered questionnaire. Furthermore, the Pakistan study also added that 57% of the medical and dental residents did not know the code of ethics of the Pakistan Medical and Dental Council, and Studies conducted in India among physicians, medical residents, and nurses reported that the knowledge of ethics ranged from 34.8% to 59% [21, 22, 24, 25]. These findings indicate the necessity of improved educational methods of medical ethics and warrant regulations to implement the practice of bioethics in the healthcare delivery system in India and other Asian countries [46–48] as well. The vast difference in the knowledge level between these studies with our study could be attributed firstly to the large sample size in this study, secondly, this study included a varied range of professionals unlike the other studies and the construct of the KAP questions varied as well. Not surprisingly, 96.9% of the participants in this study reported attending training in bioethics.

### Attitude towards bioethics

Most Maharashtra HCPs (97.1%) believed that an emotional and sexual relationship with the patient or patient’s family member is unethical and 97.6% agreed that ethical conduct is vital to avoid legal action. It is noteworthy, that these ethics in some way represent the common construct of the Indian traditional ethics, culture-related ethics, and religious opinions regarding definitive punishment for immoral acts [49]. It is difficult to explain, why most of the HCPs (80.4%) in this study opted to be neutral for performing multiple tests to avoid medical errors. This indecision or decision to take neutral stands could be because the HCPs might believe that more clinical tests are expensive, might also lead to more confusion than diagnosis, unnecessary or harmful, and not cost-effective particularly, for the low-middle economic strata population [50]. Referral of your patient to the expert physician in case of an unsure diagnosis is the key requirement of ethical medical practice. However, 35.7% of HCPs disagreed with the guidelines-based statement “If you are unsure of the diagnosis of the patient referred to you, it is better to refer the patient to an expert physician” seemingly indicating that in most of the instances where the HCPs in Maharashtra practice at rural set up where the options of referring to an expert may not be a suitable decision for example in case emergency cases and the act of childbirth [51].

Hence, it is important to explore these guideline principles in a regionally sensitive manner. Indian HCPs most often face ethical dilemmas at the rural maternal centers, and child health centers especially when buying time is not possible when advanced medical care is far from accessible in most of South Asia [52, 53]. Furthermore, the overall proportion of HCPs reporting favorable attitudes (59.4%) was on par with the studies conducted in Sri Lanka, Saudi Arabia, Manipur (India), and Pakistan [14–16, 54]. It is also noteworthy that many HCPs in this study have an opinion rather than disagree with the attitude-related bioethics guidelines. This can be explained by the inclusion of a wide range of professionals with different specialties leading to more indecisions based on their practice with different natures of ethical dilemmas and perceptions. For instance, the ethical dilemma faced by HCPs is also not alike in women's health, pediatric, end-of-life care, physical rehabilitation, dentistry, and nursing care.

### Practice of bioethics

In this study, only 34.4% of HCPs in Maharashtra reported to practice of bioethical principles either always or most often. The online survey method in contrast to face-to-face interviews would have prompted self-reporting of unethical practices or deviation from guidelines with freedom and difference in response quality [55]. On the other hand, this high proportion of bad practice of bioethics and the distribution of responses to the 10-item bioethics practice questions in this study reveals that unethical medical practice might rather be common. Only 8.8% of HCPs reported accepting the patient’s request of not willing to be examined by the interns or trainees. Though this could be justified by the scarcity of healthcare workers [56], it could also be more of clinical negligence of practice and other possible explanations could be the participation of a high proportion of academic clinicians or clinical academicians from teaching hospitals in Maharashtra, India. However, the examination of patients by the interns would most often lead to wrong diagnoses and possible medical errors in management. Medical students see medical errors differently in academic health centers. Nearly fifty percent of residents in one study had treated a patient with an adverse event [57]. Residents claimed excessive work hours, poor supervision, and poor hand-offs harm patients. Although physical examination errors can cause adverse patient events, we are unaware of any studies on medical trainee perceptions of the relationship between patient harm and inadequate clinical examination skills [58].

About 70% of the HCPs in this study, responded that getting any form of benefits from patients is unethical. Accepting gifts or other benefits from the patients is always unethical based on Indian Medical Association (IMA) ethics guidelines [3]. Irrelevant to the value or size of gifts, taking advantage of the patient's condition should be prohibited. Only 11% of the HCPs, reported that discussing ethical issues of patient care during clinical rounds, practicing equity when it is applied to resource management, and most importantly only a meager number of professionals responded that they would bring any professional misconduct to the notice of the regulatory authority. These areas were to be under consideration among the study population and demand specific attention and also deserve deliberation. This is also suggestive of the possibility of more unrevealed cases of medical misconduct not being brought to notice which could be more alarming [59].

### Factors associated with knowledge, attitude, and practice

Adequate knowledge and good/fair practice of bioethics were significantly associated with the younger age of the HCPs. This is a positive observation indicating the possibility of further improving consideration of bioethics and the prospect of good practice. HCPs with higher educational attainment were associated with both adequate knowledge and a favorable attitude, but the good practice of bioethics was not associated which suggests the possibility of unethical practice among specialists. HCPs working in the private sector and NGO were found to have good practices of bioethics, which is similar to several studies in South Asia [13, 15, 16, 20, 25] and the authors of these surveys reported there is a possibility of over-reporting of good practices of bioethics. Among the professionals the PTs and OTs are less likely to have good practice, the findings of this study are similar to the study conducted among Nigerian physiotherapists [60], the authors referred to the ethics curriculum and lower knowledge as possible reasons. HCPs who practice at Pune and Nashik are also less likely to have good practice of bioethics. HCPs with less than 15 years of experience are more likely to have good practice.

The UNESCO Chair of Bioethics Haifa at the International Center of Health, Law, and Ethics, University of Haifa, was formed in 2001 to coordinate and stimulate an International Network of Institutes for Medical Ethics Training. Recognizing the importance of UNESCO Bioethics training [61], the Indian Medical Association, National Board of Examinations, and Medical Council of India (MCI) have established bioethics units at several governmental and private deemed universities across the country [62]. The National Board of Examinations identified nodal centers and trained teachers. In 2019, the National Medical Commission of India introduced the Competency Based Curriculum in Medical Education (CBME) for undergraduate medical students nationwide with a new module called Attitude, Ethics, and Communication (AETCOM) rendering humanistic education. The AETCOM module emphasizes communication skills, ethics, professionalism, health systems beyond medical knowledge, and clinical skills. The findings of the recent studies on the effect of AETCOM on the empathy, behavioral trends, communication skills, and professional attitude of Indian medical students demonstrated favorable outcomes [63–66]. Further, integrating the AETCOM model into other allied medical healthcare curricula in India seems promising.

The mention of the study's limitations would help the readers execute caution while interpreting the findings of this study. First, the online survey method would have possibly caused over-reporting and under-reporting of certain items in the questionnaire based on the personnel bias of the respondents and social desirability bias. The responses were based on voluntary online survey participation, which might have resulted in sample selection bias and the 25% non-response rate, sensitive questions related practice of bioethics could have led to non-response and response bias. Second, reported practice might be influenced by institutional hierarchy which cannot be ruled out, the field of clinical practice was not considered and limited participation of OTs and PTs may restrict the generalizability of these findings to practitioners of a specific discipline. The other gap of this KAP-bioethics survey tool is the non-inclusion of biomedical research-related items in the construct. Finally, the cross-sectional nature of this study did not allow the determination of cause and effect relationship. Nevertheless, this study is one of the scarce surveys to report a state-level KAP of bioethics with a power-calculated sample for each region in one the largest states in India and is likely to provide a novel insight into one of the significant (bioethics) issues in the medical practice in the region. And, considering that this is a mail-based survey it is accessible to the mobile responders, this would have improved sample representativeness. The proposed clinical implications of this study are: the methods described in this study, and the KAP-bioethics survey tool will surely aid healthcare institutions in identifying potential gaps in bioethics among HCPs, further plan educational programs to address those gaps, and retest the effect of the program.

## Conclusion

In conclusion, the adequate knowledge observed among 2/3 of respondents is encouraging and would favor the laying foundation for forming a good bioethics framework. Unfortunately, the being and doing of good practice of bioethics is reported to be lacking, and the notable cases of medical negligence, unethical practice, and poor relationships between healthcare workers and patients in the region are not surprising. The findings of this study shall open up other research and serious considerations to evaluate the compliance level of bioethics practice in Maharashtra among HCPs.

### Supplementary Information

**Supplementary Material 1.** 

**Supplementary Material 2.** 

## Data Availability

The study contains all of the study data related to these findings. Requests for more information on the dataset and questions about data sharing should be directed to the correspondence author.

## Abbreviations

ADB: Asian Development Bank
AOR: Adjusted Odds Ratio
COR: Crude Odds Ratio
CI: Confidence Interval
SDG: Sustainable Development Goals
HCPs: Health Care Professionals
HCDS: Health Care Delivery System
HRH: Human Resource for Health
NCDRC: National Consumer Disputes Redressal Commission
NITI: National Institution for Transforming India
NGO: Non-Government Organization
OT: Occupational Therapists
PT: Physiotherapist
WHO: World Health Organization
