# Supplementary material for: Understanding, being, and doing of bioethics; a state-level cross-sectional study of knowledge, attitude, and practice among healthcare professionals
Source: BMC Med Ethics. 2024 Mar 18;25:30. doi: 10.1186/s12910-024-01028-w (PMC10949768; doi:10.1186/s12910-024-01028-w)
Supplement: Supplementary file 1 — Supplementary Material 1. [file 12910_2024_1028_MOESM1_ESM.docx]

Online survey questionnaire template

Knowledge, attitude, and practice of bioethics among health care professionals in Maharashtra State, India

*General instructions:*

*Please respond promptly to all the questions in the survey*

*Please pay attention and respond*

*Please tick only one suitable response for each question*

| **s.no** | **Domain and questions** | **Responses** | | | | | | | **Code** | |
| --- | --- | --- | --- | --- | --- | --- | --- | --- | --- | --- |
| 1. | Date of form submission | DD/MM/YYYY | | | | | | | D1 | |
| 2. | Consent read & obtained | Yes / No | | | | | | |  | |
| 3. | Time taken to fill the form | **__ __ __** minutes | | | | | | | T1 | |
| 4. | Respondent mail ID |  | | | | | | |  | |
|  | **[A] Socio-demographic and occupational characteristics** | | | | | | | |  | |
| 5. | What is your age? |  | | | | | | | SD1 | |
| 6. | Please, tick your gender | Male Female | | | | | | | SD2 | |
| 7. | Tick your current region or location of practice | 1. Konkan | | 2.Pune | | | | | SD3 | |
|  |  | 3.Nashik | | 4.Marathwada | | | | |  |  |
|  |  | 5.Vidarbha | |  | | | | |  |  |
| 8. | Please mention your profession? | 1. Medical doctor 2. Dentist  3. Physiotherapist 4. Nurse  5. Occupational therapist | | | | | | | SD4 | |
| 9. | What is your highest educational attainment? | Under graduation / Post-graduation / Super speciality | | | | | | | SD5 | |
| 10. | What is the type of your institution? | 1.Governmental 2.Private  3. Others specify________________ | | | | | | | SD6 | |
| 11. | Please select your work experience in years? | 1. 0 – 5 2) 5 – 10 3) 10 – 15   4) 15 – 20 5) > 20 | | | | | | | SD7 | |
| 12. | Have you ever received training in bioethics? | 1. Yes 2. No | | | | | | | SD8 | |
| **[B] KNOWLEDGE domain (please read the following questions carefully)** | | | | | | | | | | |
| 13. | Paternalism is not an ethical attitude because it conflicts with | 1. Patients autonomy 2. b) Doctors task and duties 3. Patients medical care 4. d) Doctor’s autonomy | | | | | | | K1 | |
| 14. | All of the following are true about the non-maleficence principle EXCEPT? | 1. Includes an obligation not to harm intentionally. 2. Includes informed consent and truth telling. 3. Physicians must refrain from providing ineffective. 4. Offers little useful guidance to physician because many beneficial therapies also have serious risks. | | | | | | | K2 | |
| 15. | Double effect in bioethics usually regarded as the combined effect of | 1. Beneficence & autonomy 2. Non-malpractice and confidentiality 3. Autonomy and Justice 4. Beneficence and Non-maleficence. | | | | | | | K3 | |
| 16. | The concept of justice in Bioethics is | 1. An obligation of the patient to the society 2. Taken as patients right to choose or refuse treatment 3. All medical professionals to do good for all patients under any circumstances 4. The health resources must be distributed according to the principles of equity | | | | | | | K4 | |
| 17. | Confidentiality can be breached | 1. When the patient does not listen to the doctor 2. When a patient authorize to do so 3. When financial resources are scarce and patient has not complaint 4. For a patient who requires invasive treatment. | | | | | | | K5 | |
| 18. | Right versus wrong may be a moral temptation, but right vs right is | 1. An ethical dilemma 2. The best of both worlds 3. A political brawl 4. A chance for correct action | | | | | | | K6 | |
| 19 | Specific code or metaprogram hard wired in human mind that can be exploited to cause harm is | 1. Autonomy 2. Vulnerability 3. Dignity 4. Justice | | | | | | | K7 | |
| 20.. | The most important advantage of an advance directive is to | 1. Include family & friends in the healthcare wishes 2. Help patients state their healthcare wishes to healthcare providers 3. Guarantee patient’s goals and wishes will be followed 4. Ensures that life support is made available to patient | | | | | | | K8 | |
| 21. | Informed consent is a | 1. Agreement to take part in research 2. Freely taken decision to participate or decline participation in a research project after careful consideration of the risks and benefits 3. Legal document similar to memorandum 4. Research Ethics Brochure | | | | | | | K9 | |
| 22. | The Institutional review board (IRB) is charged with? | 1. Conducting inquiries into research misconduct 2. Reviewing recruitment materials and strategies 3. Protecting the rights and welfare of human subjects 4. Assuring that all applicable institutional policies, regulations and statutory provisions are complied with | | | | | | | K10 | |
|  | **[C] Attitude of the participants towards bioethics** | | | | | | | |  | |
|  | ***SA – Strongly agree, A – Agree, N – Neutral, D – Disagree, SD – Strongly disagree*** | | | | | | | | | |
|  | **Questions** | | **SA** | | **A** | **N** | **D** | **SD** | |  |
| 23. | If the diagnosis of the referred patient is unsure better refer to the expert physician | |  | |  |  |  |  | | A1 |
| 24. | If patient refuse treatment due to beliefs, they should be instructed to find another healthcare professional | |  | |  |  |  |  | | A2 |
| 25. | Healthcare professional should sign and write name on the official documents relating to patient care such as laboratory and other diagnostic requests and results, prescriptions, certificates, patient records and other reports | |  | |  |  |  |  | | A3 |
| 26. | A healthcare profession can legally disclose information to protect the patient from danger | |  | |  |  |  |  | | A4 |
| 27. | Show you are paying attention to this survey questions by disagreeing to this statement? | |  | |  |  |  |  | | A5 |
| 28. | An emotional or sexual relationship with a patient (or with a member of the patient’s family), even with consent, is unethical | |  | |  |  |  |  | | A6 |
| 29. | After successful completion of research, its benefits should be shared to the society at national and international level | |  | |  |  |  |  | | A7 |
| 30. | A male healthcare professional should refuse to examine a uncomfortable female patient, If a female attendant is not available, | |  | |  |  |  |  | | A8 |
| 31. | Multiple tests can be performed to avoid a medical error | |  | |  |  |  |  | | A9 |
| 32. | The patient needs to be informed about their diagnosis even if they don’t have requisite knowledge | |  | |  |  |  |  | | A10 |
| 33. | Ethical conduct is important for avoiding legal action | |  | |  |  |  |  | | A11 |
|  |  | | | | | | | |  | |
|  | Questions | | **SA** | | **A** | **N** | **D** | **SD** | |  |
| 34. | I explain the nature, purpose, and possible consequences of treatment or procedure while obtaining informed consent from patients | |  | |  |  |  |  | | P1 |
| 35. | During clinical rounds along with clinical aspects of patients care, I discuss ethical issues also | |  | |  |  |  |  | | P2 |
| 36. | I practice equity especially when it is applied to resource management | |  | |  |  |  |  | | P3 |
| 37. | I render the same level of care to my clients in regular practice and over-time | |  | |  |  |  |  | | P4 |
| 38. | Please select ‘strongly agree’ to show you are paying attention to this question? | |  | |  |  |  |  | | P5 |
| 39. | I regularly re-examine the patients to find the effectiveness of the ongoing treatment | |  | |  |  |  |  | | P6 |
| 40. | I accept the patient’s request not to be examined by interns/trainees | |  | |  |  |  |  | | P7 |
| 41. | I listen to all the past history of the patient even if that doesn’t help the present treatment | |  | |  |  |  |  | | P8 |
| 42. | Getting monetary/gift benefits from patient’s treated by me is unethical | |  | |  |  |  |  | | P9 |
| 43. | When Health records are shared with other doctors for opinion I secure it with suitable firewall or password | |  | |  |  |  |  | | P10 |
| 44. | When I come across an instance of professional misconduct, I bring it to the notice of the regulatory authority | |  | |  |  |  |  | | P11 |

*Thank you for your valuable time and prompt response*
