# Supplementary material for: Understanding, being, and doing of bioethics; a state-level cross-sectional study of knowledge, attitude, and practice among healthcare professionals
Source: BMC Med Ethics. 2024 Mar 18;25:30. doi: 10.1186/s12910-024-01028-w (PMC10949768; doi:10.1186/s12910-024-01028-w)
Supplement: Supplementary file 2 — Supplementary Material 2. [file 12910_2024_1028_MOESM2_ESM.docx]

**Additional tables**

**Table S1 - Internal consistency of the knowledge, attitude, practice in bioethics (KAP-Bioethics) scale (n = 25).**

| **Dimensions** | **Items, n** | **Score, mean (SD)** | **Cronbach α** |
| --- | --- | --- | --- |
| Knowledge (K) | 10 | 6.7 (1.21) | 0.73 |
| Attitude (A) | 10 | 24.7 (10.13) | 0.80 |
| Practice (P) | 10 | 28.6 (5.1) | 0.81 |

**Table S2 - Test-retest reliability (ICC), mean, standard deviation (SD), and standard error (SE) of mean of the KAP-bioethics tool (n = 25)**

| **Item** | **Mean (±)T1** | **SE** | **Mean (±)T2** | **SE** | **ICC (95% CI)** |
| --- | --- | --- | --- | --- | --- |
| K1 | 0.64 (0.25) | 0.032 | 0.68 (0.41) | 0.03 | 0.71** (0.55, 0.86) |
| K2 | 0.65 (0.26) | 0.040 | 0.67 (0.29) | 0.02 | 0.77* (0.57, 0.82) |
| K3 | 0.72 (0.14) | 0.036 | 0.69 (0.39) | 0.03 | 0.70** (0.39, 0.71) |
| K4 | 0.64 (0.24) | 0.027 | 0.73 (0.48) | 0.04 | 0.71* (0.42, 0.91) |
| K5 | 0.57 (0.49) | 0.04 | 0.67 (0.43) | 0.04 | 0.74** (0.41, 0.84) |
| K6 | 0.58 (0.30) | 0.04 | 0.66 (0.37) | 0.03 | 0.76**(0.40, 0.89) |
| K7 | 0.57 (0.31) | 0.04 | 0.61 (0.28) | 0.03 | 0.75**(0.47, 0.77) |
| K8 | 0.70 (0.20) | 0.02 | 0.69 (0.19) | 0.02 | 0.70** (0.40, 0.78) |
| K9 | 0.44 (0.50) | 0.04 | 0.48 (0.48) | 0.04 | 0.71* (0.51, 0.88) |
| K10 | 0.79 (0.41) | 0.03 | 0.68 (0.27) | 0.02 | 0.71*(0.42, 0.77) |
| A1 | 2.01 (1.0) | 0.09 | 2.1 (0.91) | 0.09 | 0.72**(0.59, 0.82) |
| A2 | 2.10 (1.1) | 0.09 | 2.22 (1.0) | 0.05 | 0.74**(0.50, 0.79) |
| A3 | 2.0 (1.2) | 0.08 | 2.09 (0.81) | 0.10 | 0.76**(0.58, 0.70) |
| A4 | 2.69 (1.3) | 0.11 | 2.74 (0.98) | 0.11 | 0.73**(0.56, 0.80) |
| A6 | 2.21 (1.1) | 0.10 | 2.30 (0.91) | 0.10 | 0.74**(0.46, 80) |
| A7 | 1.97 (1.3) | 0.19 | 2.32 (0.78) | 0.10 | 0.77**(0.29, 0.80) |
| A8 | 2.77 (0.99) | 0.10 | 2.64 (0.92) | 0.11 | 0.74**(0.49, 0.85) |
| A9 | 2.97 (1.2) | 0.09 | 2.86 (0.85) | 0.09 | 0.77**(0.38, 0.97) |
| A10 | 2.87 (1.3) | 0.10 | 2.80 (0.94) | 0.07 | 0.76**(0.52, 0.96) |
| A11 | 2.80 (1.2) | 0.11 | 2.79 (0.69) | 0.06 | 0.79*(0.34, 0.92) |
| P1 | 2.99 (0.71) | 0.05 | 3.01 (0.66) | 0.07 | 0.70**(0.51, 0.87) |
| P2 | 2.61 (0.81) | 0.07 | 2.62 (1.2) | 0.07 | 0.70*(0.29, 0.81) |
| P3 | 2..41 (0.69) | 0.06 | 2.39 (0.68) | 0.06 | 0.84**(0.61, 0.92) |
| P4 | 2.55 (0.87) | 0.05 | 2.60 (0.9) | 0.06 | 0.73**(0.24, 0.74) |
| P6 | 3.01 (0.92) | 0.07 | 3.23 (1..41) | 0.08 | 0.71*(0.26, 0.86) |
| P7 | 2.70 (0.79) | 0.07 | 2.70 (1.3) | 0.07 | 0.71**(0.44, 0.93) |
| P8 | 2.52 (0.87) | 0.08 | 2.59 (0.90) | 0.08 | 0.73**(0.39, 0.91) |
| P9 | 2.69 (0.86) | 0.07 | 3.10 (0.89) | 0.05 | 0.72*(0.30, 0.87) |
| P10 | 3.2 (0.78) | 0.05 | 3.14 (0.73) | 0.08 | 0.71* (0.22, 0.89) |
| P11 | 2.74 (0.62) | 0.04 | 2.56 (0.72) | 0.06 | 0.72**(0.31, 0.90) |

SE – Standard error, ± - Standard deviation, T1 – test one, T2 – retest, items A15 and P26 are attention check items hence not included in the analysis. **indicates significance at the level of < 0.001 and * indicates significance at the level of < 0.01
